# Supplementary material for: Evolution of the Ergot Alkaloid Biosynthetic Gene Cluster Results in Divergent Mycotoxin Profiles in Claviceps purpurea Sclerotia
Source: Toxins (Basel). 2021 Dec 2;13(12):861. doi: 10.3390/toxins13120861 (PMC8704706; doi:10.3390/toxins13120861)
Supplement: Supplementary file 1 [file toxins-13-00861-s001.zip › toxins-1458610-supplementary.pdf]

Supplementary Materials: Evolution of the ergot alkaloid biosynthetic gene cluster results in divergent mycotoxin profiles in *Claviceps purpurea* sclerotia

Carmen Hicks, Thomas E. Witte, Amanda Sproule, Tiah Lee, Parivash Shoukouhi, Zlatko Popovic, Jim G. Menzies, Christopher N Boddy, Miao Liu, David P. Overy

Table S1. Origin of *C. purpurea* Isolates.

Isolate	Identification		Host	Designation	Collection Year	Province
LM04	*Cl. purpurea s.s.*	G1	*Tricosecale*	DAOMC250624	1996	MB
LM30	*Cl. purpurea s.s.*	G1	*Secale cereale*	DAOMC250649	2000	SK
LM60	*Cl. purpurea s.s.*	G1	*Avena sativa*	DAOMC250680	2005	MB
LM207	*Cl. purpurea s.s.*	G1	*Elymus repens*	-	2014	MB
LM232	*Cl. purpurea s.s.*	G1	*Phalaris canariensis*	DAOMC250822	2014	MB
LM233	*Cl. purpurea s.s.*	G1	*Phalaris canariensis*	-	2014	MB
LM469	*Cl. purpurea s.s.*	G1	*Triticum aestivum*	-	2016	ON
LM474	*Cl. purpurea s.s.*	G1	*Hordeum vulgare*	-	2016	ON

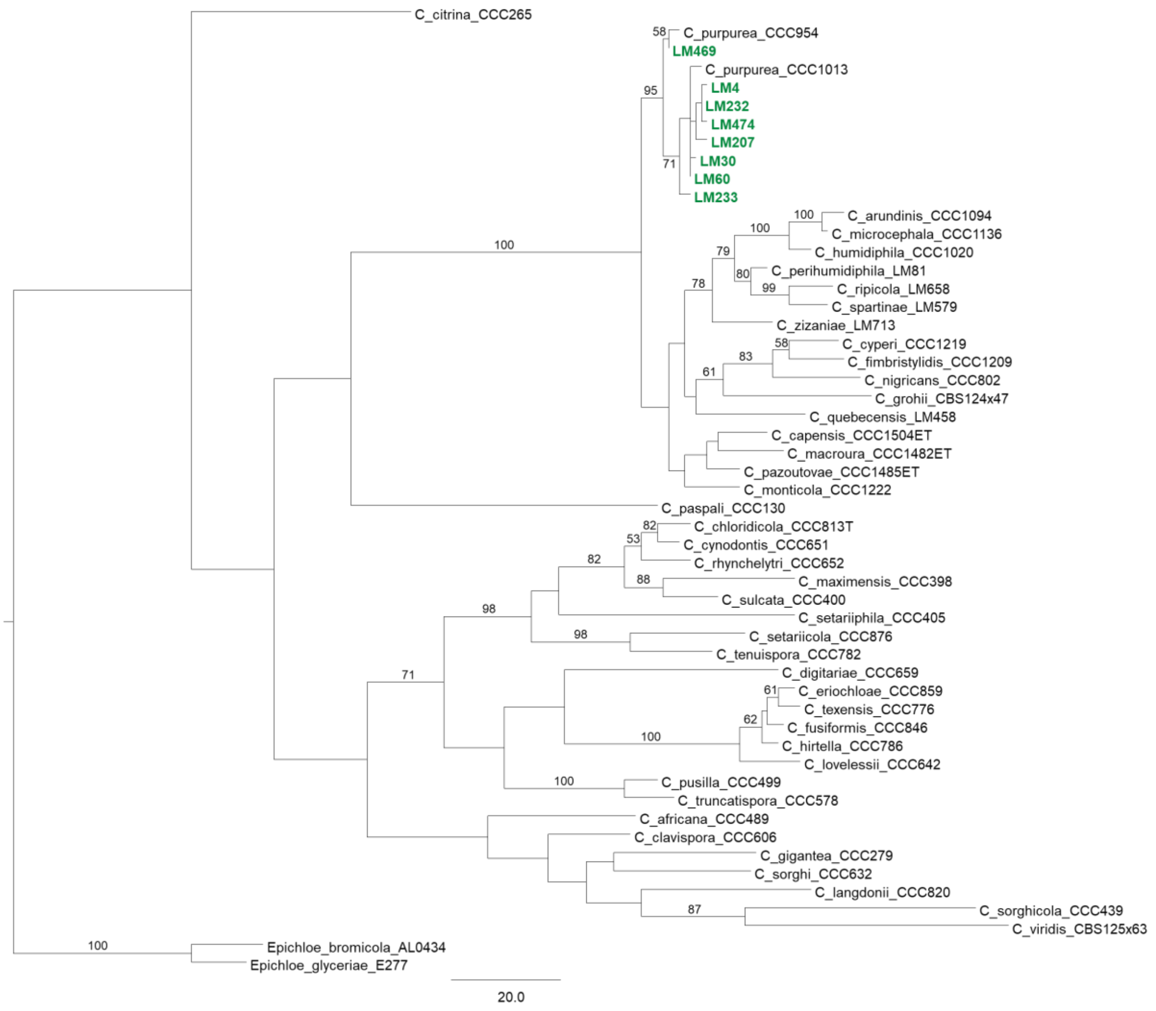

Figure S1. Concatenated *RPB2* & *TEF1–α* gene sequence MP (maximum parsimony) algorithm phylogenetic analyses of 44 *Claviceps* spp. 8 isolates used in analysis (green) cluster within in the *C. purpurea* clade.

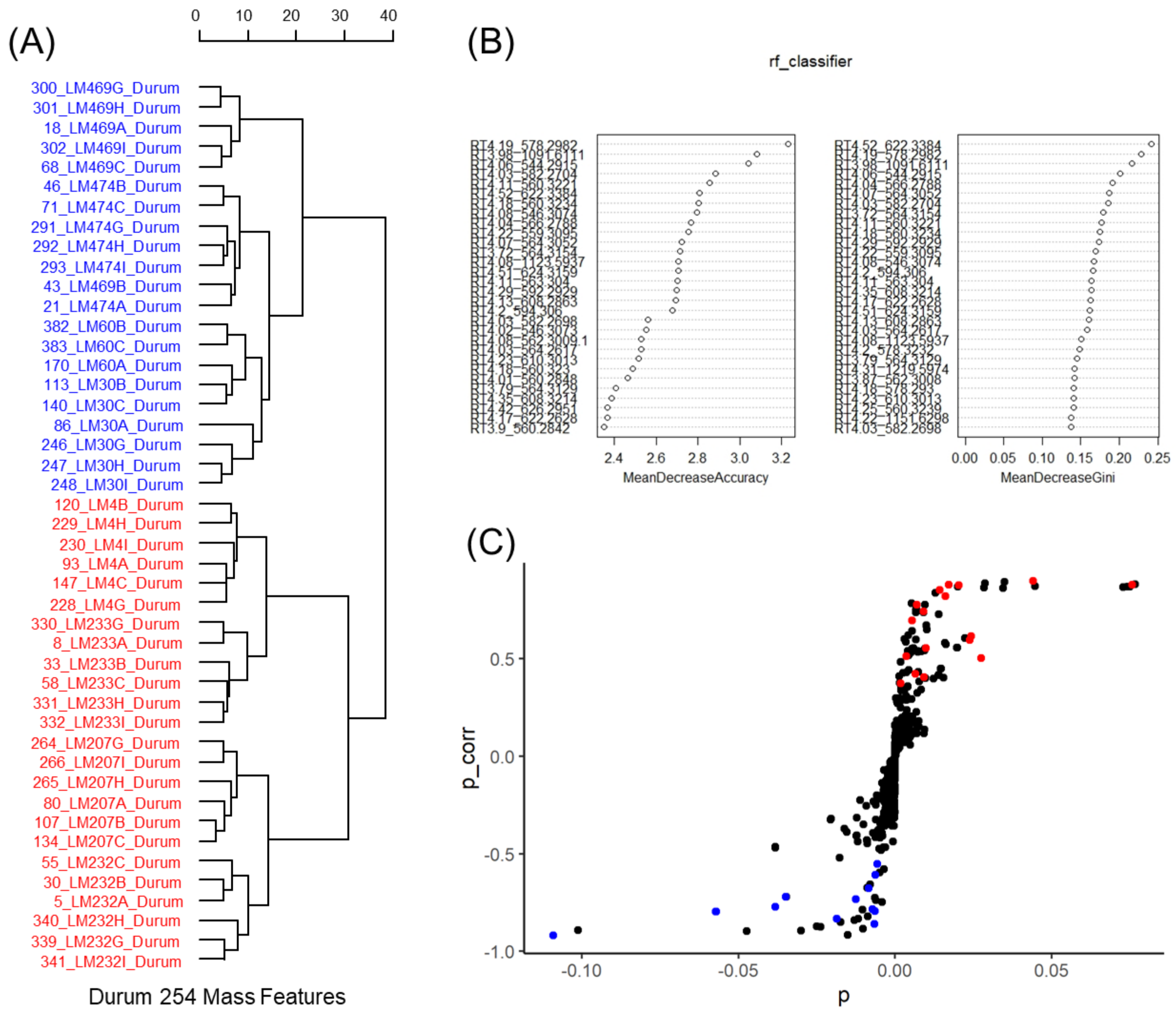

Figure S2: Untargeted metabolomics analysis of *C. purpurea* strains grown on Durum wheat. (**A**) Euclidean Ward hierarchical clustering of pseudo-binary (presence/absence) mass feature data of replicate sclerotia. Labels in Red and Blue represent specimen identified in Class 1 and Class 2 respectively with (n=6) replicates per specimen. (**B**) Top 30 mass features associated with class formation as determined via random forest analysis of raw data (Left: Mean decrease in accuracy and Right: Mean decrease in Gini of raw data of replicate sclerotia). (**C**) S-plot from OPLS-DA highlighting top 30 features identified by random forest analysis of replicate sclerotia, features in red and blue represent specimen identified in Class 1 and Class 2.

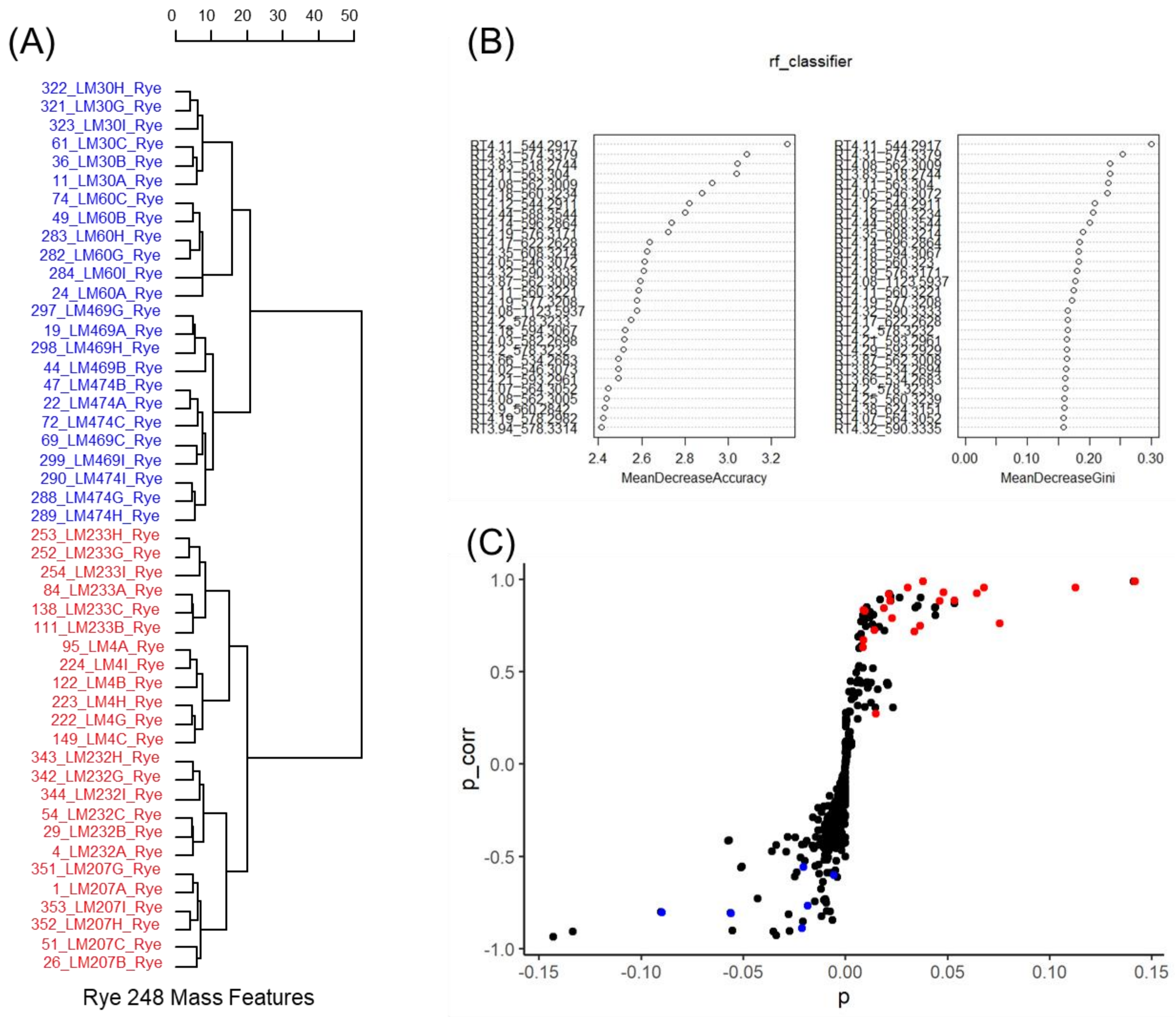

Figure S3: Untargeted metabolomics analysis of *C. purpurea* strains grown on rye. (**A**) Euclidean Ward hierarchical clustering of pseudo-binary (presence/absence) mass feature data of replicate sclerotia. Labels in Red and Blue represent specimen identified in Class 1 and Class 2 respectively with (n=6) replicates per specimen. (**B**) Top 30 mass features associated with class formation as determined via random forest analysis of raw data (Left: Mean decrease in accuracy and Right: Mean decrease in Gini of raw data of replicate sclerotia). (**C**) S-plot from OPLS-DA highlighting top 30 features identified by random forest analysis of replicate sclerotia, features in red and blue represent specimen identified in Class 1 and Class 2.

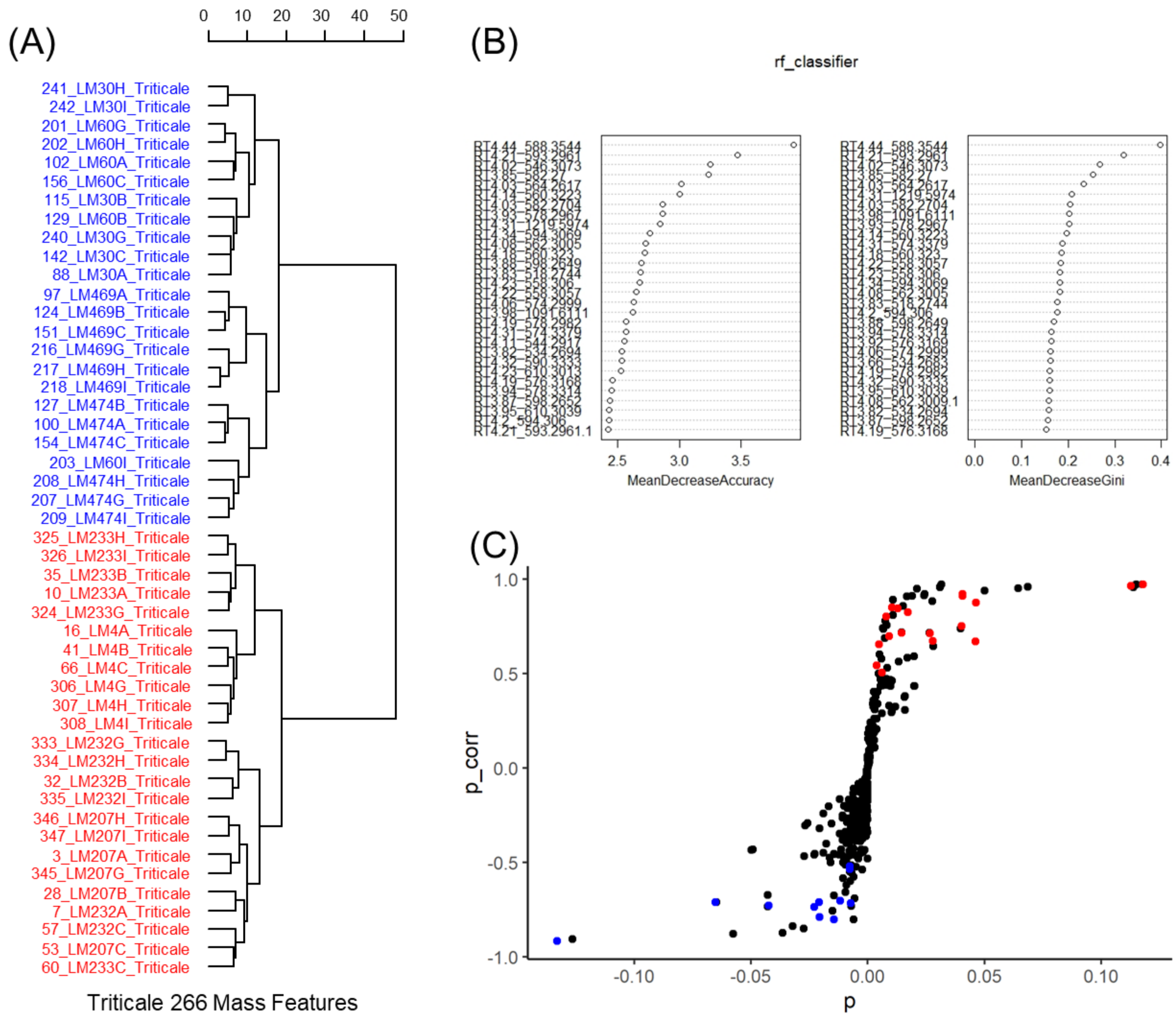

Figure S4: Untargeted metabolomics analysis of *C. purpurea* strains grown on triticale. (**A**) Euclidean Ward hierarchical clustering of pseudo-binary (presence/absence) mass feature data of replicate sclerotia. Labels in Red and Blue represent specimen identified in Class 1 and Class 2 respectively with (n=6) replicates per specimen. (**B**) Top 30 mass features associated with class formation as determined via random forest analysis of raw data (Left: Mean decrease in accuracy and Right: Mean decrease in Gini of raw data of replicate sclerotia). (**C**) S-plot from OPLS-DA highlighting top 30 features identified by random forest analysis of replicate sclerotia, features in red and blue represent specimen identified in Class 1 and Class 2.

Table S2: Putative ergot alkaloid $[M+H]^+$ annotations of ergot alkaloid $[M+H]^+$ mass features

Retention Time (min)	Observed $[M+H]^+$ *m/z*	Theoretical [M+H]+ *m/z*	Δ(ppm)	Formula	Putative Annotation
2.94	269.1279	269.1284	1.8578	$C_{16}H_{17}N_2O_2$	Lysergic Acid Derivative
3.03	326.1852	326.1862	3.0657	$C_{19}H_{24}N_3O_2$	Ergometrine
3.20	271.1435	271.1440	1.8440	$C_{16}H_{19}N_2O_2$	Simple Clavine
3.22	257.1645	257.1648	1.1666	$C_{16}H_{21}N_2O$	Simple Clavine
3.30	257.1645	257.1648	1.1666	$C_{16}H_{21}N_2O$	Simple Clavine
3.34	326.1853	326.1862	2.7592	$C_{19}H_{24}N_3O_2$	Ergometrinine
3.37	257.1645	257.1648	1.1666	$C_{16}H_{21}N_2O$	Simple Clavine
3.68	548.2860	548.2867	1.2767	$C_{30}H_{38}N_5O_5$	Isobaric w/ Ergosine
3.72	564.3154	564.3180	4.6073	$C_{31}H_{41}N_5O_5$	Dihydroergocornine
3.78	548.2864	548.2867	0.5472	$C_{30}H_{38}N_5O_5$	Isobaric w/ Ergosine
3.82	534.2694	534.2710	2.9947	$C_{29}H_{36}N_5O_5$	Ergovaline
3.85	582.2700	582.2710	1.7174	$C_{33}H_{36}N_5O_5$	Isobaric w/ Ergotamine
3.87	562.3008	562.3023	2.6676	$C_{31}H_{40}N_5O_5$	Ergocornine
3.88	598.2649	598.2659	1.6715	$C_{33}H_{35}N_5O_6$	Hydroxyergotamine
3.92	562.3004	562.3023	3.3790	$C_{31}H_{40}N_5O_5$	Isobaric w/ Ergocornine
3.92	576.3169	576.3180	1.9087	$C_{32}H_{42}N_5O_5$	Isobaric w/ Ergocryptine
3.94	578.3314	578.3336	3.8040	$C_{32}H_{43}N_5O_5$	Dihydroergocryptine
3.95	548.2866	548.2867	0.1824	$C_{30}H_{38}N_5O_5$	Ergosine
3.95	610.3039	610.3023	-2.6217	$C_{35}H_{40}N_5O_5$	Isobaric w/ Ergocristine
3.96	532.2913	532.2924	2.0665	$C_{30}H_{38}N_5O_4$	Ergosam
3.98	273.1600	273.1597	-1.0983	$C_{16}H_{21}N_2O_2$	Simple Clavine
4.01	268.1435	268.1444	3.3564	$C_{16}H_{18}N_3O$	Lysergic Acid Derivative
4.02	546.3073	546.3074	0.1830	$C_{31}H_{40}N_5O_4$	Ergocornam
4.03	582.2704	582.2710	1.0304	$C_{33}H_{36}N_5O_5$	Ergotamine
4.04	566.2788	566.2767	-3.7084	$C_{33}H_{36}N_5O_4$	Ergotamam
4.08	546.3074	546.3074	0.0000	$C_{31}H_{40}N_5O_4$	Isobaric w/ Ergocornam
4.08	562.3005	562.3023	3.2011	$C_{31}H_{40}N_5O_5$	Isobaric w/ Ergocornine
4.11	560.3221	560.3231	1.7847	$C_{32}H_{42}N_5O_4$	Isobaric w/ Ergocryptam
4.14	560.3223	560.3231	1.4277	$C_{32}H_{42}N_5O_4$	Isobaric w/ Ergocryptam
4.14	596.2864	596.2867	0.5031	$C_{34}H_{38}N_5O_5$	Ergostine
4.18	560.3230	560.3231	0.1785	$C_{32}H_{42}N_5O_4$	Isobaric w/ Ergocryptam
4.19	576.3171	576.3180	1.5616	$C_{32}H_{42}N_5O_5$	Ergocryptine
4.20	594.3060	594.3074	2.3557	$C_{35}H_{40}N_5O_4$	Ergocristam
4.24	610.3014	610.3023	1.4747	$C_{35}H_{40}N_5O_5$	Ergocristine
4.25	560.3239	560.3231	-1.4277	$C_{32}H_{42}N_5O_4$	Isobaric w/ Ergocryptam
4.25	574.3381	574.3387	1.0447	$C_{33}H_{44}N_5O_4$	Isobaric w/ Ergoannam

4.31	574.3379	574.3387	1.3929	$C_{33}H_{44}N_5O_4$	Ergoannam
4.32	590.3335	590.3336	0.1694	$C_{33}H_{44}N_5O_5$	Ergogaline
4.34	594.3069	594.3074	0.8413	$C_{35}H_{40}N_5O_4$	Isobaric w/ Ergocristam
4.35	608.3214	608.3231	2.7946	$C_{36}H_{42}N_5O_4$	Ergosedmam
4.38	624.3151	624.3180	4.6451	$C_{36}H_{42}N_5O_5$	Ergosedmine
4.51	624.3159	624.3180	3.3637	$C_{36}H_{42}N_5O_5$	Ergosedminine

Table S3: Stachelhaus codes and predicted substrate specifics for all lpsA genes extracted from long-read genomes. * NRPSpredictor2 (Röttig et al. 2011) was unable to predict the specificity of certain codons. In these cases, the nearest neighbour to the Stachelhaus code-based phylogeny is written in brackets, along with the number of codon sites consistent with this prediction (max 10/10). Highlighted codon residues are potentially contributing to predicted substrate specificity changes.

Gene/domain	Strain	Positions										Predictions
		235	236	239	278	299	301	322	330	331	517	NRPSpred2*
lpsA1/aa1	LM04	D	A	I	F	C	G	G	P	L	K	ala
	Cpurp20.1	D	A	I	F	C	G	G	P	L	K	ala
	LM60	D	A	I	F	C	G	G	P	L	K	ala
	LM72	D	A	I	F	C	G	G	P	L	K	ala
lpsA1/aa2	LM04	D	L	A	G	V	G	A	I	I	K	(leu 6/10)
	Cpurp20.1	D	L	V	G	M	A	A	V	G	K	phe
	LM60	D	L	V	G	M	A	A	V	G	K	phe
	LM72	D	L	V	G	M	A	A	V	G	K	phe
lpsA1/aa3	LM04	D	I	T	L	V	A	G	L	I	K	pro
	Cpurp20.1	D	I	T	L	V	A	G	L	I	K	pro
	LM60	D	I	T	L	V	A	G	L	I	K	pro
	LM72	D	I	T	L	V	A	G	L	I	K	pro
lpsA2/aa1	LM04	D	L	F	F	C	G	G	P	L	K	ala
	Cpurp20.1	D	A	V	F	C	V	G	P	A	K	ala
	LM60	D	A	I	F	C	G	G	P	L	K	ala
	LM72	D	L	F	F	C	G	G	P	L	K	ala
lpsA2/aa2	LM04	D	L	A	G	M	G	A	V	A	K	phe
	Cpurp20.1	D	L	A	G	M	G	A	M	I	K	(pro 5/10)
	LM60	D	L	A	G	M	G	A	V	A	K	phe
	LM72	D	L	A	G	M	G	A	V	A	K	phe
lpsA2/aa3	LM04	D	I	T	L	V	A	G	L	I	K	pro

Cpurp20.1	D	I	T	L	V	A	G	L	I	K	pro
LM60	D	I	T	L	V	A	G	L	I	K	pro
LM72	D	I	T	L	V	A	G	L	I	K	pro

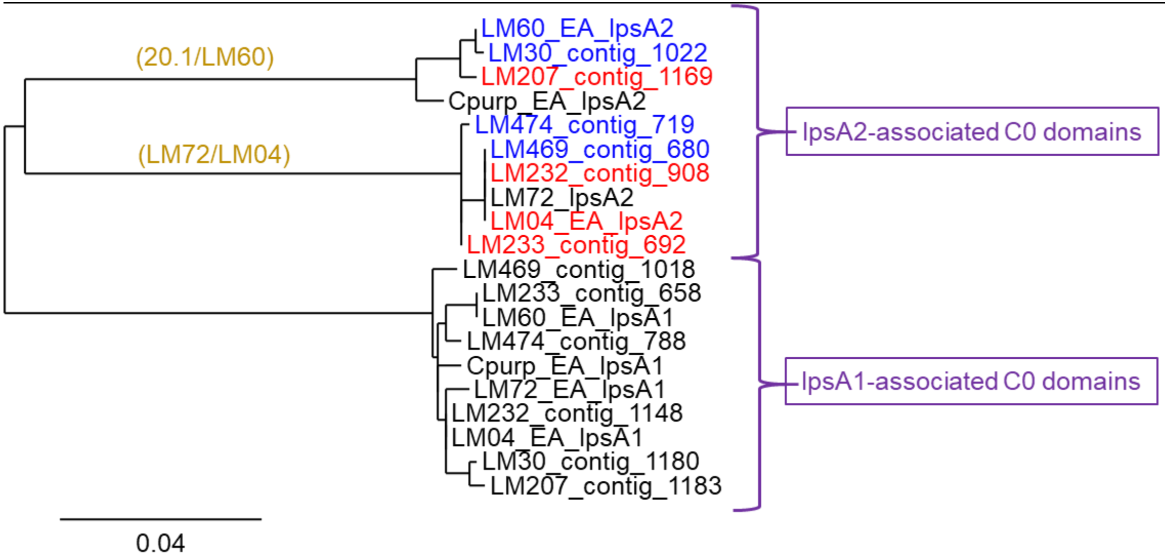

Figure S5: Phylogenetic tree of aligned nucleotides from C0 domains, with associated intergenic 'genotypes' overlaid onto branches (brown text in brackets). Genotypes are here defined as representing the highly polymorphic regions between *lpsA1* and *lpsA2*, forming two groupings either belonging to the *C. purpurea* 20.1 / LM60 strains, or as LM72 / LM04 strains, as described in Figure 7. C0 domain sequences were extracted from blast hits of the LM04 *lpsA1* C0 domain queried against all genomes used in this study. Sequences were aligned, curated and visualized using the phylogeny.fr 'one click' web portal (accessed September 24th, 2021). C0 domains belonging to the two putative lpsA2 groupings are approximately 85% identical to each other, and both are approximately 83-84% similar to the monophyletic lpsA1 C0 domains. LpsA2-associated leaves are coloured red or blue depending on the metabolomic class associated with the strain (red = class 1, blue = class 2).
